# Current status and future aspects in the Japan Stroke Data Bank

**DOI:** 10.3389/fneur.2023.1090136

**Published:** 2023-03-22

**Authors:** Shinichi Wada, Sohei Yoshimura, Kaori Miwa, Yoshitaka Iwanaga, Masatoshi Koga, Kazunori Toyoda

**Affiliations:** ^1^Department of Information and Health, National Cerebral and Cardiovascular Center, Suita, Osaka, Japan; ^2^Department of Cerebrovascular Medicine, National Cerebral and Cardiovascular Center, Suita, Osaka, Japan

**Keywords:** Japan Stroke Data Bank, cerebrovascular disease, cardiovascular disease, stroke registry, acute ischemic stroke, intracerebral hemorrhage, subarachnoid hemorrhage

## Abstract

The Japanese National Plan for the Promotion of Measures Against Cerebrovascular and Cardiovascular Diseases was formulated on October 27, 2020. One purpose of this plan was to promote research on cerebrovascular and cardiovascular diseases. Therefore, it is necessary to clarify the actual status of stroke treatment in Japan and operate a national stroke database with high public interest completely and accurately. The Japan Stroke Data Bank (JSDB; https://strokedatabank.ncvc.go.jp/en/) was established by the Ministry of Health, Labor and Welfare Scientific Research in Shimane University (Shimane, Japan) in 1999 and was transferred to the National Cerebral and Cardiovascular Center (Osaka, Japan) as a part of the Cardiovascular Disease Registry in 2015. More than 200,000 of stroke cases have been registered using individual forms from more than 100 nationwide stroke centers over ~20 years. Since there are few large-scale stroke registries with nationwide coverage in Asia, including Japan, compared with those in Europe and North America, the role of the JSDB in the plan will be important in the future. To construct a high-quality stroke registry, we aimed to (1) collect detailed data through individual questionnaires for each participating stroke center, (2) link to external databases (e.g., insurance claims and public death registries), (3) improve the quality of treatment at participating hospitals through benchmarking, and (4) obtain stable funding through sustained support from government and academic societies. We also describe the history of the JSDB and changes in the trend of real-world stroke treatment in Japan based on the results of analysis of data in the JSDB.

## 1. Introduction

Stroke is the fourth leading cause of death and requires the most nursing care in Japan (~30% of all diseases) (1). Since the annual medical cost of stroke is estimated at ~1.7 trillion yen and the cost of nursing care at ~1.9 trillion yen (2), the effect of stroke on the society and economy of Japan is extremely high. Thus, in December 2018, the Cerebrovascular and Cardiovascular Disease Control Act was established, which was the first legislative measures to stroke and cardiovascular disease in Japan (3). The Japanese National Plan for Promotion of Measures Against Cerebrovascular and Cardiovascular Diseases was also formulated on October 27, 2020 (4). The plan included preventive measures and dissemination of accurate information for cerebrovascular and cardiovascular diseases, and development of service systems on medicine, health and welfare. One of the objectives of the plan was to promote research on cerebrovascular and cardiovascular diseases. Therefore, it was necessary to establish and maintain a comprehensive and accurate nationwide database and serve the public interest by promoting rational and economical stroke countermeasures.

**Figure 1:**
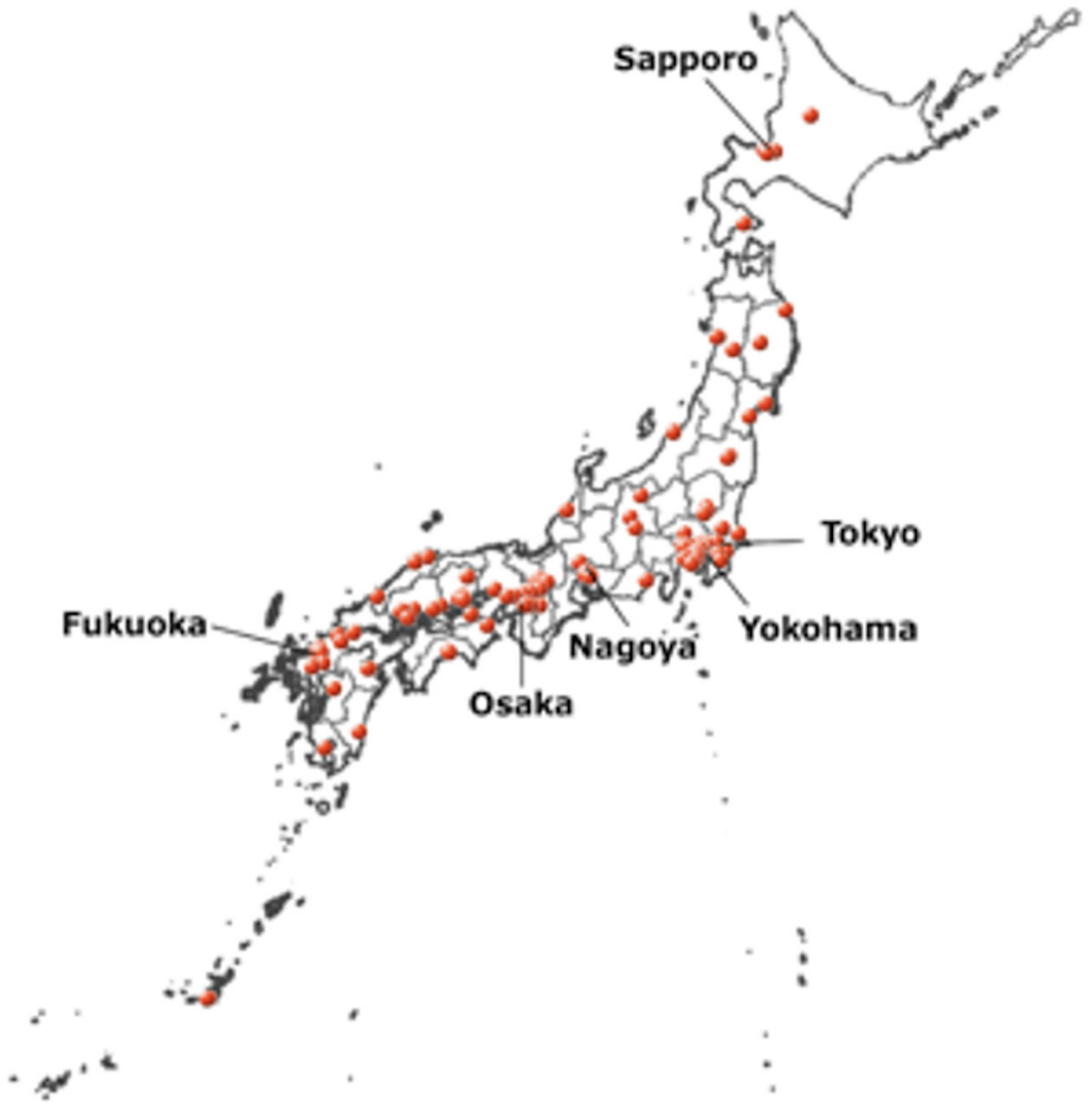
Distribution of hospitals participating to the Japan Stroke Data Bank. The hospitals were shown in white map with red points, and the map were proceeded by the authors using GSI map (6) https://maps.gsi.go.jp/vector/#4/35.354185/131.992306/&ls=vblank&disp=1&d=l.

The Japan Stroke Data Bank (JSDB), a Japanese stroke registry started from 1999, has been collecting clinical data using individual patient data, including patients' characteristics, examination, treatment, and stroke outcomes, from many hospitals in Japan (5). As of July 2021, 132 hospitals participated in the JSDB, and approximately 240,000 patients with acute ischemic stroke (AIS), intracerebral hemorrhage (ICH), subarachnoid hemorrhage (SAH), and transient ischemic attack (TIA) have been registered (Figure 1) (6). The JSDB can provide information for the construction of a nationwide database in the future. In this review, we report about the history and recent results analyzed by the JSDB.

## 2. History of the JSDB

The prototype of the JSDB was a research project started in 1999 by Shotai Kobayashi (currently Professor Emeritus, Shimane University) to construct a database of patients with acute stroke (including TIA) under a Grant-in-Aid for Scientific Research. The project management was transferred from Shimane University (Shimane, Japan) to the National Cerebral and Cardiovascular Center (Osaka, Japan) in 2015.

In 2016, a 5-year plan to overcome stroke and cardiovascular diseases was published in collaboration with the Japan Stroke Association and Japan Cardiovascular Society. The plan listed the promotion of registration projects as a part of its major goals, which emphasized the need for a nationwide stroke patient registry system. Thus, research groups were organized under the auspices of the Japan Agency for Medical Research and Development (FY2015, FY2016, granted for Kazuo Minematsu) to develop a system. The group validated domestic and international stroke registry studies, for example “European Registers of Stroke” and “Get With the Guidelines-Stroke,” to propose an optimal medical information collection system for Japan (7–9). Consequently, several points were identified. First, there are few large and well-disseminated registry studies in Asia, including Japan, compared with those in Europe. Second, more patients were registered using opt-out methods or were not required to give informed consent. Third, no stroke registry was identified in Japan with support from academic societies and patient advocacy groups then, in addition to public research funds such as the International Stroke Registry. Fourth, cooperation with other databases using personal IDs also progressed in Europe. Therefore, a stroke registry meeting the following four goals needed to be established: (1) collecting detailed data through individual questionnaires for stroke centers, (2) linking to external databases (e.g., insurance claims and public death registries), (3) improving the quality of treatment at participating hospitals through benchmarking, and (4) obtaining stable funding through sustained support from governments and academic societies. The JSDB, one of the stroke registries in Japan, aims to achieve these four goals.

## 3. Location of the JSDB

The JSBD, similar to many stroke registries, uses individual questionnaires to collect data. The use of individual questionnaires allows setting of items according to a specific purpose and ensures collection of detailed information; however, it is labor intensive, particularly in inputting and cleaning data. For this reason, many countries are attempting to manage registries to obtain accurate data while reducing the burden on participants by using web system or a dedicated web application (7). As an example of stroke registries using individual questionnaires in Asia, the China National Stroke Registry is a government-led hospital-based registry that enrolls more than 10,000 patients annually with detailed individual data collection and 2-year follow-up (10).

In Japan, there are regional stroke registries that collect individual data, such as the Akita Stroke Registry, Fukuoka Stroke Registry, and Takashima Stroke Registry (11–13). However, other than the JSDB, no other stroke registries are known to collect data using individual questionnaires across Japan. As a nationwide stroke registry in Japan, the J-ASPECT Study is currently active and has successfully obtained a large amount of data in a short period using Diagnosis Procedure Combination (DPC) data (14). DPC is developed as a measurement tool for transparency in the content of acute inpatient care, with the aim of standardizing, evaluating and improving the quality of medical care in Japan (15). DPC data are extracted from external sources, enabling collection of a large amount of patient information using existing large-scale data. Moreover, once the extraction method is established, information can be collected continuously with less effort in terms of complete coverage and continuity. Meanwhile, the JSDB aims to build a database that will aggregate individual data from all over Japan and enable comprehensive and more detailed analysis by adding information not included in the DPC.

## 4. Management system of the JSDB

The Department of Cerebrovascular and Cardiovascular Disease Information (currently the Department of Medical and Health Information Management) serves as the secretariat under the management of a steering committee comprising a group of stroke medical researchers from across Japan. We have started to modify the management system to obtain stable funding through sustained support from the government and academic societies. In detail, we prepared a research protocol that complied with current research ethics policies. We do not obtain written consent from the patients; however, we provide an opt-out opportunity for patients who do not wish to use their information because it is important to ensure complete coverage to achieve high academic and social importance. Next, we adopted a web-based collection system using a multipurpose clinical data repository system (16). In the past, data were collected using standalone PCs and sent periodically, but this system allows the secretariat to manage the system, review data, and clean data as needed. The survey items are updated as appropriate according to current stroke trends. Data are collected at the National Cerebral and Cardiovascular Center of the Department of Medical and Health Information Management. In the future, a low-labor, low-cost, highly comprehensive, and accurate information collection system needs to be established for each hospital. We would also like to link the JSDB with existing large-scale databases in the future.

## 5. Publications from the JSDB

**Figure 2:**
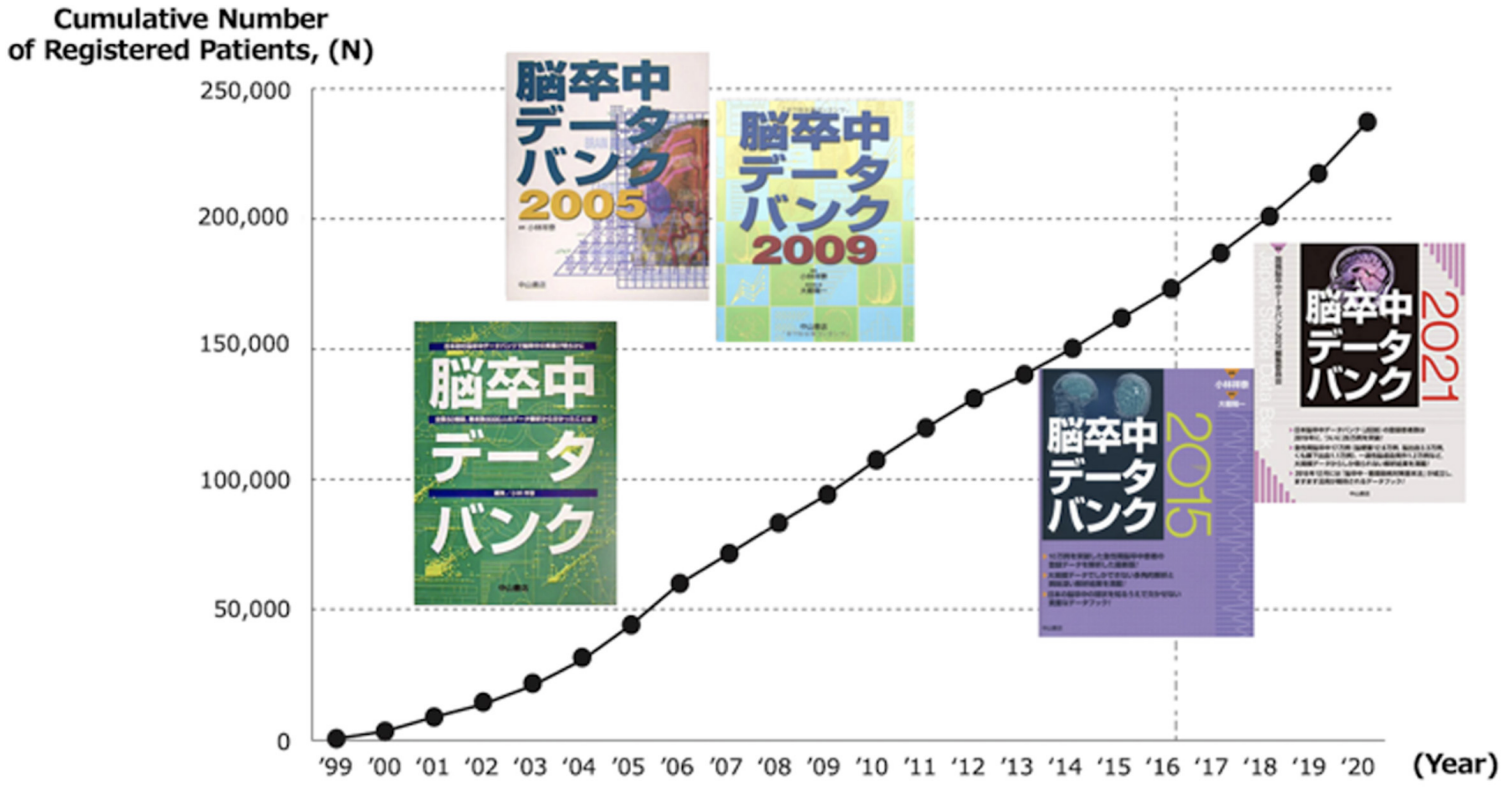
Cumulative number of registered patients and outcome books. Created based on Figure 1, Page 2 in (17) © 2021 Nakayama Shoten.

**Figure 3:**
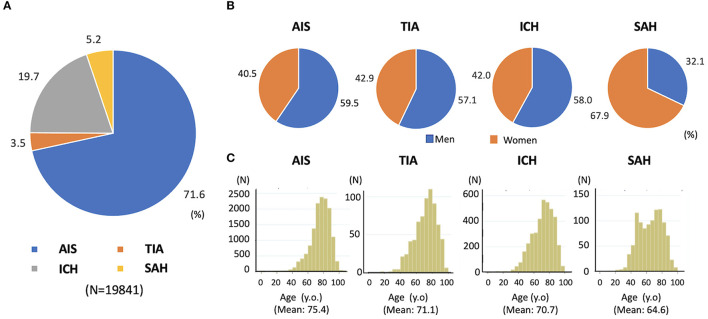
Distribution of stroke subtype, sex and age in 19841 patients registered in JSDB during 2020 year. **(A)** Stroke subtypes, **(B)** sex, and **(C)** age. AIS, acute ischemic stroke; ICH, intracerebral hemorrhage; JSDB, Japan Stroke Data Bank; SAH, subarachnoid hemorrhage; TIA, transient ischemic attack. The data was cited with modification from annual report of JSDB in 2021. (Available from reference 5, in Japanese).

The collected data, including baseline characteristics, examination, treatment, and stroke outcome, were published regularly. The JSDB data were published in Japanese as five volumes set at intervals of approximately 5 years from 2003 by Nakayama Shoten Co., Ltd., (Figure 2) (17). In these series, researchers from the participating hospitals reported the results of analyzed data on major issues related to acute stroke. In addition, the collected data were analyzed at the National Cardiovascular Disease Information Center every year, targeting data from patients registered within the previous year. The results were sent to the participating hospitals once a year as an annual report and made publicly available on the web in Japanese (5). Figure 3 showed a part of annual report in patients registered in 2020. If others want to use the collected data for academic purposes, such as reprinting or citations in academic papers, the source can be cited clearly. For example, the annual report of the JSDB has been cited in papers on malignancy-related strokes and post-stroke discharge destinations (18, 19). In addition to academic purposes, researchers can request the data by applying to the secretariat and specifying the purpose and intended use of the report without payment. Permission is granted if the use is considered highly public, such as for stroke awareness activities in the mass media. We aim to continue and expand the scale of patient information collection, enhance academic activities, provide feedback to participating hospitals, and provide information to public institutions, academic societies, and public interest groups, further promoting information disclosure and returning research results to society.

## 6. Recent reports published by the JSDB

Several reports have recently been published using JSDB-collected data. For example, changes in stroke severity and outcomes within 7 days after stroke onset, including ischemic and hemorrhagic stroke, for the past 20 years from January 2000 to December 2019 in the JSDB have been reported (20). Briefly, 183,082 patients (135,268 patients with AIS, 36,014 patients with ICH, and 11,800 with SAH) were examined. Women accounted for 39.8, 42.7, and 67.2% of cases with a median age of 74 (66–82), 70 (59–79), and 64 (53–75) years in AIS, ICH, and SAH, respectively. The National Institutes of Health (NIH) Stroke Scale score on admission was a median of 5 [2–13]/3 [2–8] (women/men, respectively) for AIS patients and 12 [4–24]/11 [4–22] for ICH patients. The median value of the World Federation of Neurosurgical Surgeons (WFNS) score in SAH was 2 [1–5]/2 [1–4]. In AIS patients, 8.6/8.9% of patients received reperfusion therapy. The median modified Rankin Scale (mRS) score at discharge was 3 [1–4]/2 [1–4] in AIS patients, 4 [2–5]/4 [2–5] in ICH patients, and 3 [0–5]/2 [0–5] in SAH patients. The rate of in-hospital death was 6.1/3.6% for AIS, 13.9/14.9% for ICH, and 22.1/20.9% for SAH.

This study demonstrated that the median onset age increased in all the three types of stroke, and the NIH Stroke Scale score in AIS and ICH and the WFNS score in SAH decreased during the past 20 years on multivariable analysis. Moreover, although the rate of the favorable outcome (mRS 0–2 at discharge) of AIS patients increased over time after adjustment for age in both sexes, it decreased after adjusting for reperfusion therapy, especially in men, which may reflect the efficacy of reperfusion therapy (Table 1). In contrast, the rate of the favorable outcome of ICH and SAH patients did not increase over time in the multivariable analysis, suggesting the lack of a powerful therapy equivalent to reperfusion therapy in AIS or the widespread use of anticoagulant agents prior to stroke onset. Thus, the long study duration and large population in the JSDB helped to clarify the trend of stroke outcomes in Japan.

**Table 1:** Secular changes in favorable outcomes at discharge.

	**Odds ratio (95% CI)^a^**			
**Outcome**	**Crude**	**Model 1^b^**	**Model 2^c^**	**Model 3^d^**
**Women**				
Total ischemic stroke	0.994 (0.995–1.003)	1.020 (1.015–1.024)	1.003 (0.998–1.009)	0.997 (0.991–1.003)
Cardioembolism	1.009 (1.002–1.017)	1.037 (1.029–1.045)	1.023 (1.012–1.034)	1.008 (0.997–1.019)
Large-artery atherosclerosis	1.010 (1.003–1.018)	1.028 (1.020–1.036)	1.004 (0.994–1.014)	1.002 (0.992–1.013)
Small-vessel occlusion	0.997 (0.989–1.005)	1.014 (1.005–1.022)	0.986 (0.975–0.997)	0.985 (0.974–0.995)
Intracerebral hemorrhage	0.984 (0.976–0.992)	0.994 (0.986–1.003)	0.980 (0.968–0.992)	NA
Subarachnoid hemorrhage	1.000 (0.990–1.010)	1.011 (1.000–1.022)	1.002 (0.989–1.016)	NA
**Men**				
Total ischemic stroke	1.002 (0.999–1.005)	1.015 (1.011–1.018)	0.995 (0.991–1.000)	0.990 (0.985–0.994)
Cardioembolism	1.006 (1.000–1.013)	1.023 (1.016–1.029)	1.007 (0.998–1.016)	0.993 (0.984–1.002)
Large-artery atherosclerosis	1.009 (1.003–1.015)	1.020 (1.014–1.026)	1.001 (0.993–1.008)	0.998 (0.991–1.006)
Small-vessel occlusion	0.997 (0.991–1.004)	1.009 (1.002–1.016)	0.982 (0.973–0.991)	0.980 (0.971–0.989)
Intracerebral hemorrhage	0.983 (0.976–0.990)	0.989 (0.982–0.996)	0.971 (0.961–0.982)	NA
Subarachnoid hemorrhage	0.996 (0.982–1.009)	1.002 (0.988–1.017)	0.989 (0.970–1.008)	NA

Abbreviations: NA, not applicable; NIHSS, National Institutes of Health Stroke Scale; WFNS, World Federation of Neurological Surgeons.

a Odds ratio (95% CI) per 1 year.

b Model 1 is adjusted by age.

c Model 2 is adjusted by age, NIHSS score (WFNS grade for subarachnoid hemorrhage), and history of stroke.

d Model 3 is adjusted by age, NIHSS score (WFNS grade for subarachnoid hemorrhage), history of stroke, and reperfusion therapy.

The table is cited from Table 2 in (20) [Toyoda, et al. JAMA Neurol. (2022) 79:61–69].

The other study in JSDB has shown that the effect impact of renal dysfunction on stroke onset and outcomes differed according to the clinical pathology (21). In detail, 10,392 AIS patients whose results of serums creatinine level or dipstick proteinuria on admission were available were examined. Among patients with AIS, lower estimated glomerular filtration rate (eGFR) levels were associated with a higher rate in cardioembolic stroke and lower in small vessel occlusion linearly (21). As for unfavorable outcome (mRS 3–6 at discharge), lower eGFR <40 mL/min/1.73 m^2^ was significantly associated in both small vessel occlusion and cardioembolic stroke. In addition, since higher eGFR was also significantly associated with unfavorable outcomes in cardioembolic stroke, eGFR had U-shaped association to the outcomes in cardioembolic stroke (21). Moreover, proteinuria (dipstick proteinuria ≥1) was associated with unfavorable outcome in patients with both stroke subtypes on multivariable analysis (Odds-ratio 3.18 [95% confidence interval: 2.03–4.98] in cardioembolic stroke and 2.08 [1.08–3.98] in small vessel occlusion, respectively). Chronic kidney disease is known to enhance endothelial dysfunction, various atherosclerotic change including calcification and alternations in coagulation systems resulted in a prothrombotic state, which lead to increased risk of any AIS and to an associated worse outcome. This study demonstrates that renal impairment was associated with difference in distributions and outcomes between cardioembolic stroke and small vessel occlusion and may have a predictive value for outcome after specific AIS subtypes.

We are also actively disseminating information worldwide by publishing papers using data from this project, such as the association between habitual alcohol consumption and stroke severity and the creation of a score for the indication of treatment for ruptured cerebral aneurysms (22, 23).

## 7. Conclusion

Although the promotion of registration projects in the Second 5-Year Plan to Conquer Stroke and Cardiovascular Disease is important for future stroke and cardiovascular disease countermeasures, basic all-inclusive registry data on stroke in Japan are currently lacking. Over the past 20 years, the JSDB has gradually developed and has the possibility of fulfilling its role as a large-scale depository of clinical statistics in Japan. The JSDB is expected to play a role in the registration system and serve as a cornerstone of stroke care in Japan.

## Author contributions

Study concept and design: SW and SY. Supervision: KM, YI, MK, and KT. All authors contributed to the article and approved the submitted version.
